# Supplementary material for: Adaptations in gut Bacteroidales facilitate stable co-existence with their lytic bacteriophages
Source: Gut Microbes. 2025 May 23;17(1):2507775. doi: 10.1080/19490976.2025.2507775 (PMC12118408; doi:10.1080/19490976.2025.2507775)
Supplement: Supplementary Figures.docx [file KGMI_A_2507775_SM8144.docx]

**Supplementary Figures**

**Figure S1.** Enumeration of bacteria and phage during the 5-day co-culture experiment testing a range of multiplicities of infection (MOIs) of phage (0.001 – 10) at the start of the test. After each 24-hour period, a new subculture (SC) was performed in fresh media at a dilution of 1:50. **(A)** *B. intestinalis* + ɸcrAss001, **(B)** *B. xylanisolvens* + ɸcrAss002, **(C)** *P. distasonis* + ɸPDS1, **(D)** *B. thetaiotaomicron* Δcps + DAC15. The black line represents bacterial counts (CFU/mL) for the control condition (uninfected bacteria), the blue lines represent bacterial counts (CFU/mL) in co-culture with phage at different MOIs (lighter lines indicate lower MOIs), and the red lines represent the phage counts (pfu/mL or copies/mL) in co-culture with bacteria at different MOIs (lighter lines indicate lower MOIs). Differences in phage enumeration methods were due to the ability to produce countable plaques (CFU/mL) or, alternatively, qPCR (copies/mL) when plaques were not detected.

**Figure S2.** Kinetics of bacterial growth during 24 hours after 5 days of co-culture, measured by optical density (OD_595_). **(A)** *B. intestinalis*, **(B)** *B. xylanisolvens*, **(C)** *P. distasonis*, **(D)** *B. thetaiotaomicron* Δcps. The orange line represents OD_595_ of control bacterial cultures after 5 days of subculturing, the blue line represents OD_595_ of infected bacterial cultures after 5 days of subculturing, and the black line represents the blank (growth media only, FAB). Measurements were carried out in triplicate. *: Significant differences in OD_595_ when comparing the control with the infected condition determined at *p* < 0.05.

**Figure S3.** Representation of the capsule polysaccharide (CPS) operons and gene clusters, which showed significant changes in expression after phage infection. **(A)** *B. intestinalis*, **(B)** *B. xylanisolvens* and **(C)** *P. distasonis*.

**Figure S4.** Functional classification of the proteins encoded by differentially expressed genes identified in RNA-seq analysis using the Cluster of Orthologous Groups (COGs) database. The classification and categorisation of protein functions represented are based on genes with expression changes that were located outside of CPS loci. Each graph represents the percentage distribution of protein functions according to COG categories with known functions. **(A)** *B. intestinalis*, **(B)** *B. xylanisolvens*, **(C)** *P. distasonis*, **(D)** *B. thetaiotaomicron* Δcps.

**Figure S5.** Phase-variable region and structural inversion of CPS1 in *B. xylanisolvens*. **(A)** Structure of the phase-variable operon encoding CPS1 in *B. xylanisolvens*, with each gene represented as an arrow coloured according to the gene product, as indicated in the legend. The location of the invertible promoter is also shown. **(B)** The intergenic region of the CPS1 operon shows an inversion when long-read sequencing data are compared to the reference genome. The blue area represents regions of perfect alignment, while the red area indicates inversely aligned sequences. **(C)** Close-up view of the intergenic region where the inversion occurs, displaying the nucleotide sequence involved in this structural variation (GTTACTTCTTAGGTAACGGA).

**
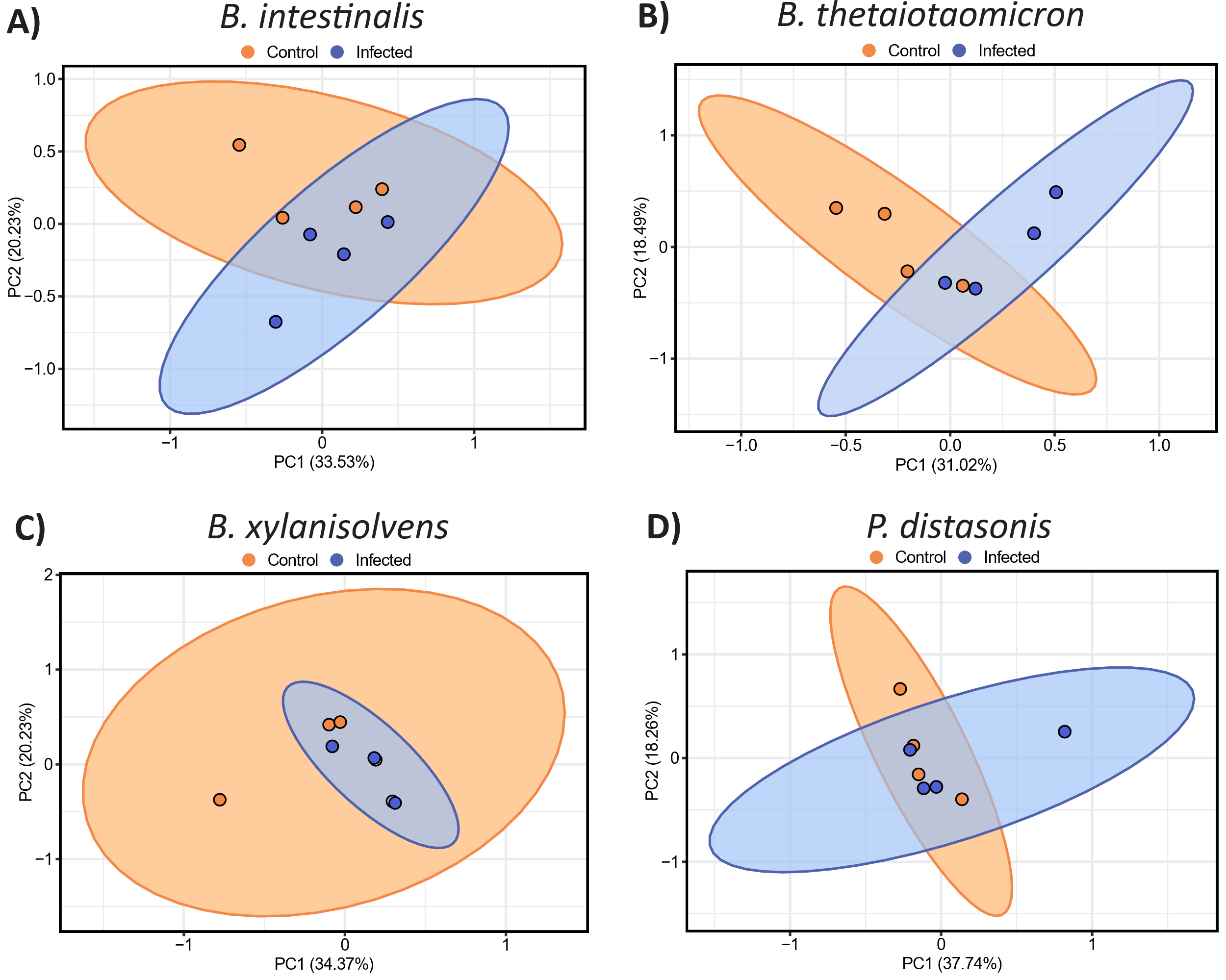
****Figure S6.** Principal Component Analysis (PCA) of proteomic profiles obtained from the pellet during the mid-log phase on the fifth day of the co-culture experiment. The orange colour represents the control condition (uninfected), while blue is the infected condition. Ellipses represent 95% confidence intervals, and the explained variances of the two principal components are shown in brackets along the axes. **(A)** *B. intestinalis* + ɸcrAss001, **(B)** *B. thetaiotaomicron* Δcps + DAC15, **(C)** *B. xylanisolvens* + ɸcrAss002, **(D)** *P. distasonis* + ɸPDS1.

**
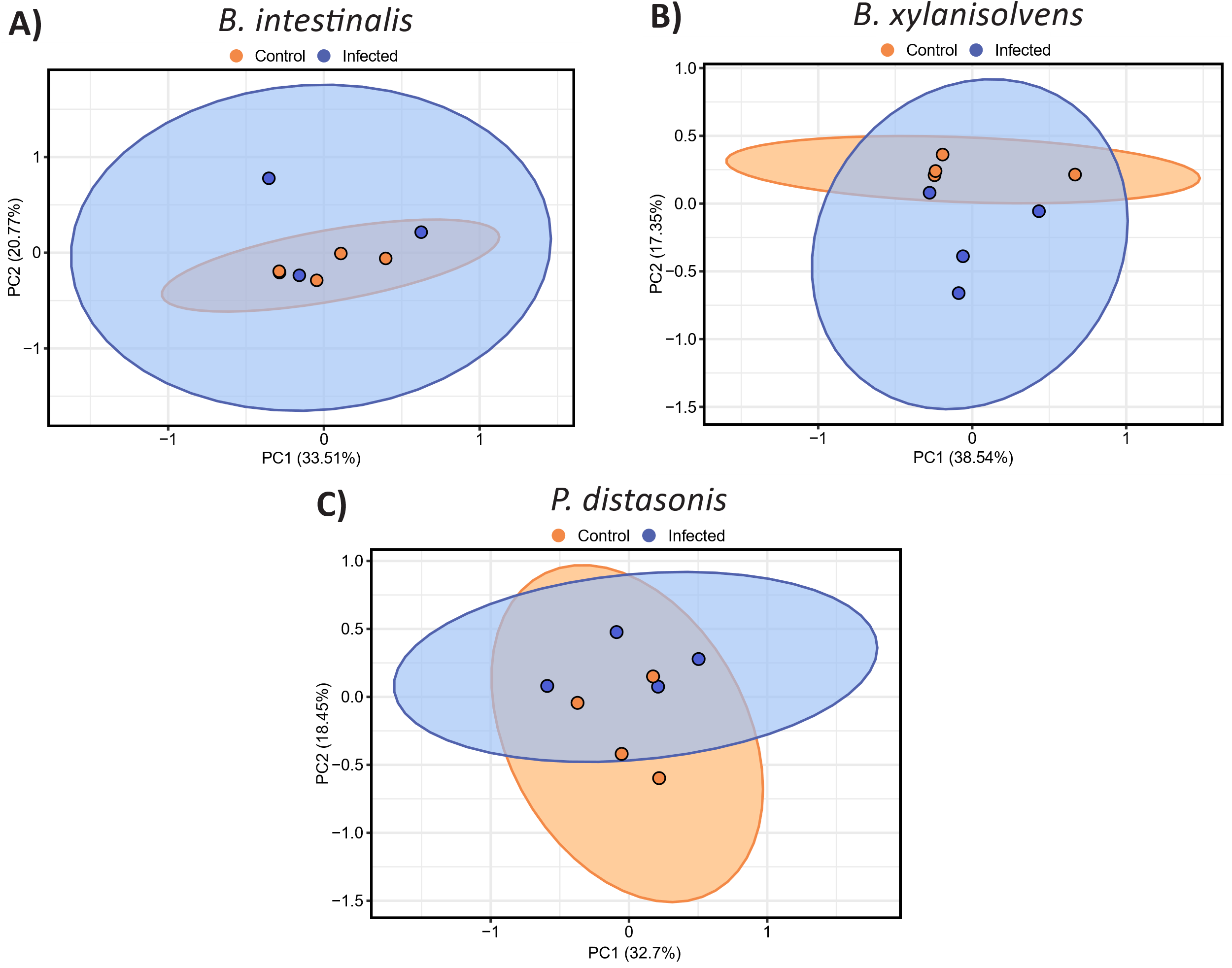
**

**Figure S7.** Principal Component Analysis (PCA) of proteomic profiles obtained from the supernatant during the mid-log phase on the fifth day of the co-culture experiment in capsular bacterial strains. Orange colour represents the control condition (uninfected), while blue represents the infected condition. Ellipses represent 95% confidence intervals, and the explained variances of the two principal components are shown in brackets along the axes. **(A)** *B. intestinalis* + ɸcrAss001, **(B)** *B. xylanisolvens* + ɸcrAss002, **(C)** *P. distasonis* + ɸPDS1.

**Figure S8.** Principal Component Analysis (PCA) of intracellular metabolomic profiles of detected compounds with accuracy levels 1 and 2a, extracted from the bacterial pellets at the mid-log phase on the fifth day of the co-culture experiment. Orange represents the control condition (uninfected), while blue is the infected condition. Ellipses represent 95% confidence intervals, and the explained variances of the two principal components are shown in brackets along the axes. **(A)** *B. intestinalis* + ɸcrAss001, **(B)** *B. xylanisolvens* + ɸcrAss002, **(C)** *P. distasonis* + ɸPDS1.
